# Upregulation of Barrel GABAergic Neurons Is Associated with Cross-Modal Plasticity in Olfactory Deficit

**DOI:** 10.1371/journal.pone.0013736

**Published:** 2010-10-29

**Authors:** Hong Ni, Li Huang, Na Chen, Fengyu Zhang, Dongbo Liu, Ming Ge, Sudong Guan, Yan Zhu, Jin-Hui Wang

**Affiliations:** 1 Department of Physiology, Bengbu Medical College, Bengbu, Anhui, China; 2 State Key Lab for Brain and Cognitive Sciences, Institute of Biophysics, Chinese Academy of Sciences, Beijing, China; Emory University, United States of America

## Abstract

**Background:**

Loss of a sensory function is often followed by the hypersensitivity of other modalities in mammals, which secures them well-awareness to environmental changes. Cellular and molecular mechanisms underlying cross-modal sensory plasticity remain to be documented.

**Methodology/Principal Findings:**

Multidisciplinary approaches, such as electrophysiology, behavioral task and immunohistochemistry, were used to examine the involvement of specific types of neurons in cross-modal plasticity. We have established a mouse model that olfactory deficit leads to a whisking upregulation, and studied how GABAergic neurons are involved in this cross-modal plasticity. In the meantime of inducing whisker tactile hypersensitivity, the olfactory injury recruits more GABAergic neurons and their fine processes in the barrel cortex, as well as upregulates their capacity of encoding action potentials. The hyperpolarization driven by inhibitory inputs strengthens the encoding ability of their target cells.

**Conclusion/Significance:**

The upregulation of GABAergic neurons and the functional enhancement of neuronal networks may play an important role in cross-modal sensory plasticity. This finding provides the clues for developing therapeutic approaches to help sensory recovery and substitution.

## Introduction

Human beings with the loss of a sensory function appear hypersensitive to other stimuli, such as blindness individuals demonstrate the enhanced touch and auditory functions for spatial identification, and deaf ones are alert to visual input for their communications [1], [2], [3], [4], [5], [6]. In these types of cross-modal sensory plasticity, the hypersensitivity in the remained sensory modalities and subsequent sensory substitution maintain the homeostasis in sensory functions and well-awareness to living environments. The elucidation of the mechanisms underlying cross-modal sensory plasticity provides the clues for developing therapeutic approaches to help sensory recovery and substitution.

Cross-modal sensory plasticity in rodents is accompanied by the enlargement of cortical areas for remained modalities [3], [5], [7], [8], the high expression of certain genes [9], [10] and the rewire/crosswire of neural circuits [7]. It is not known about its cellular mechanisms. Furthermore, is the cross-modal plasticity present in all types of sensations? In order to address these questions, we have developed a mouse model of olfactory deficit, and examined plastic changes in whisker tactile sensation and barrel cortical GABAergic neurons. A rationale for studying GABAergic neurons is that their rhythmic activities coordinate the behaviors of principal neurons in neural network [11], [12], [13], [14], [15], [16], [17]. The associative up-regulations in whisker tactile and barrel GABAergic neurons are found after a loss of olfaction.

## Results

In the studies of cross-modal plasticity and its cell-specific mechanisms, we established a mouse model of olfaction deficit by injuring olfactory epithelia on left side, and examined whether the loss of olfaction up-regulates whisker tactile sensation and induces cellular changes in barrel cortex. A week after injury of olfactory epithelia, we analyzed the behavior of whiskers and the morphology/function of GABAergic neurons in barrels. As whisker afferents project to barrel cortex on contralateral side [18] and olfactory afferents mainly input to piriform cortex on the same side [19], we presented “deprivation” in figures for the right side of whiskers and the left side of barrel cortex, whereas “control” for the left side of whiskers and the right one of barrel cortex.

### A loss of olfaction up-regulates whisker tactile sensation

**Figure 1:**
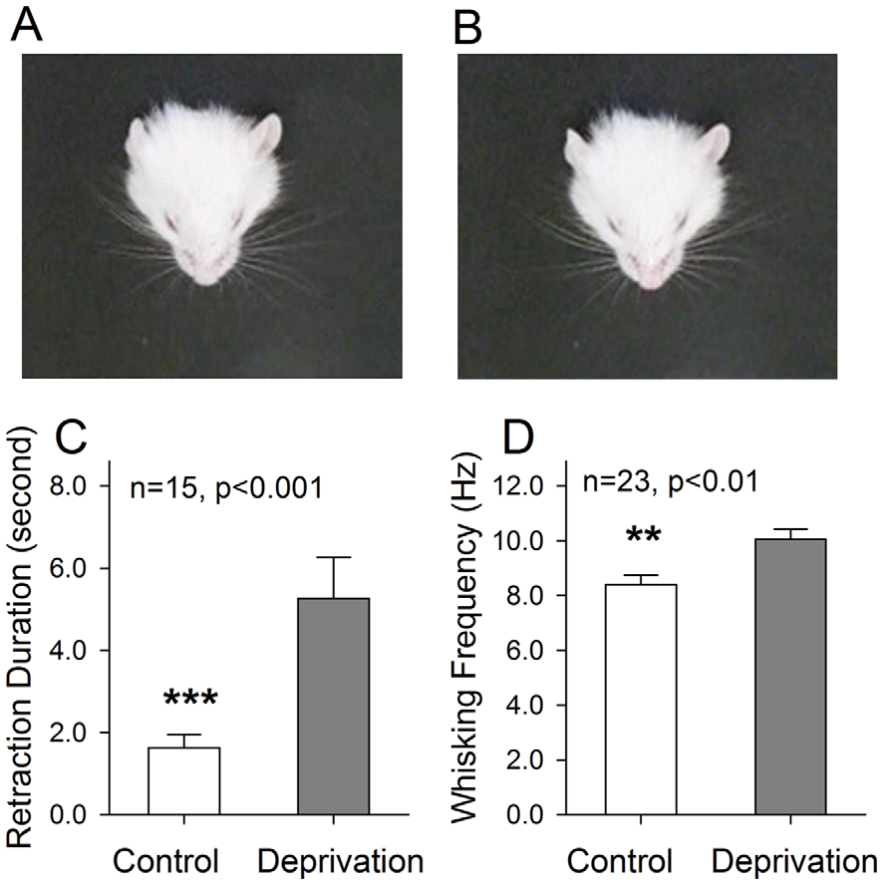
Olfactory deprivation in left nasal cavity up-regulates whisker tactile sensation in mice. **A-B**) show the retraction of whiskers induced by air-puffing toward the right side (A) and left side (B) of whiskers two seconds after air-puffing, in which the retractions are pointed by yellow arrows. **C**) shows the retraction duration of right-side whiskers (an opposite side of olfactory deprivation, gray bar) and of left-side (white) after air-puffing (n = 15, p<0.001, ***). **D**) shows the frequency of free-air whisking from right-side whiskers (gray) and left-side (white, n = 23, p<0.01, **).

The active and rhythmic sweeping of whiskers (i.e., whisking) signifies whisker tactile sensation in rodent [20], [21], [22]. The measurement of whisker behaviors usually includes free-air whisking and stimulation-induced whisker protraction- retraction [23]. To detect whisker tactile sensation, we measured the frequency of free-air whisking, which denotes active tactile sensation, and the duration of whiskers' retraction after puffing air toward them, which stands for tactile sensitivity to stimulations (Fig. 1). The correlation of whisking strength to tactile sensitivity is based on a rule in physiological reflex that the magnitude of reaction is proportional to the sensitivity of sensory system under a given stimulation.

Panels A∼B in Fig. 1 show whisker retraction induced by puffing air to control side (right in A) and olfaction-deprived side (left in B) in a mouse. Whiskers' retraction (pointed by yellow arrows) is more obvious on the right side where whisker afferents project to the left side of barrel cortex (i.e., the side of olfactory deprivation). Fig, 1C shows that the duration of whisker retraction is statistically longer in right side (gray bar) than left side (white; n = 15, p<0.001). Fig. 1D shows the comparison of whisking frequency in right side (gray bar) vs. left side (white; n = 23, p<0.01). Whisker hyperactivity associated with olfaction deficit denotes that the loss of olfactory function up-regulates whisker tactile sensation, a novel model of cross-modal sensory plasticity.

To understand cellular mechanisms underlying this cross-modal plasticity from olfactory deficit to whisker tactile upregulation, we examined changes in the morphology and functions of GABAergic neurons in barrel cortex. A rationale for this study is that the rhythmic activities of GABAergic cells coordinate the behaviors of principal neurons in neural network [14], [15], [16], [17], [24], [25].

### Anatomical changes of barrel GABAergic neurons after olfaction loss

We analyzed the number and structure of barrel cortical GABAergic cells in cross-modal sensory plasticity in FVB-Tg(GADGFP)4570Swn/J mice with olfactory injury in that somatostain-positive GABAergic cells were labeled by GFP. A week after olfactory deficit, the number of GABA cells was counted in each cross-section of barrels. The primary processes (branches from soma) and secondary ones (branches from primaries) of GABAergic neurons were measured in each of barrel sections.

**Figure 2:**
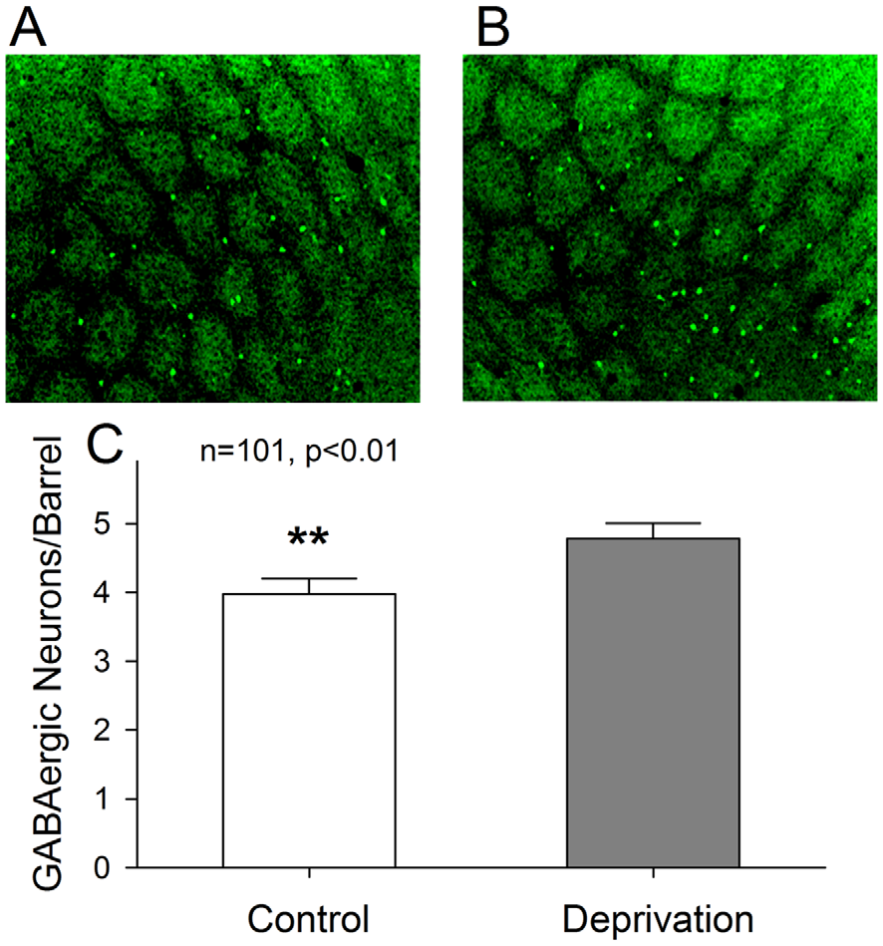
Olfactory deprivation (left nasal cavity) raises the number of barrel GABAergic cells that are somatostatin-positive and labeled with eGFP. **A**) shows a cross-section view of barrel cortex in right side (control). **B**) shows cross-section view of barrel cortex in left side (olfactory deprivation). **C**) Bar graph illustrates the number of GABAergic neurons per barrel in left side (olfaction deprivation, gray) and right (control, white; n = 101, p<0.01, **).

Fig. 2 illustrates the effects of olfaction deficit on the number of GABAergic neurons in barrel cortex. The number of GFP-labeled cells per barrel is lower in control (2A) than olfaction-deprived side (2B). Their number on the average from all optically visible barrels with clear edge, in which the number of barrels is 101 from seven mice, is 4.78±0.23 in left side (olfactory deprivation, gray bar in Fig. 2C) and 3.97±0.23 in right (control, white bar; p<0.01). Therefore, olfactory deprivation recruits more GABAergic neurons in barrel cortex.

In quantifying all subtypes of GABAergic neurons, we applied an alternative way. Cytochrome Oxidase (CO) in mitochondria is endogenous metabolic maker for neuronal activity [26], [27]. Highly active GABAergic cells consume much energy produced from CO-mediated reactions [28]. The neurons with high CO level were counted as GABAergic cells. The immunocytochemical staining of GAD67 and CO was used to be sure that CO-positive cells are GABAergic identity. As all cells are stained by hematoxylin that labels rough endoplasmic reticulum, we calculate CO-positive cells versus Nissl's cells to quantify the ratio of GABAergic neurons to total cells.

**Figure 3:**
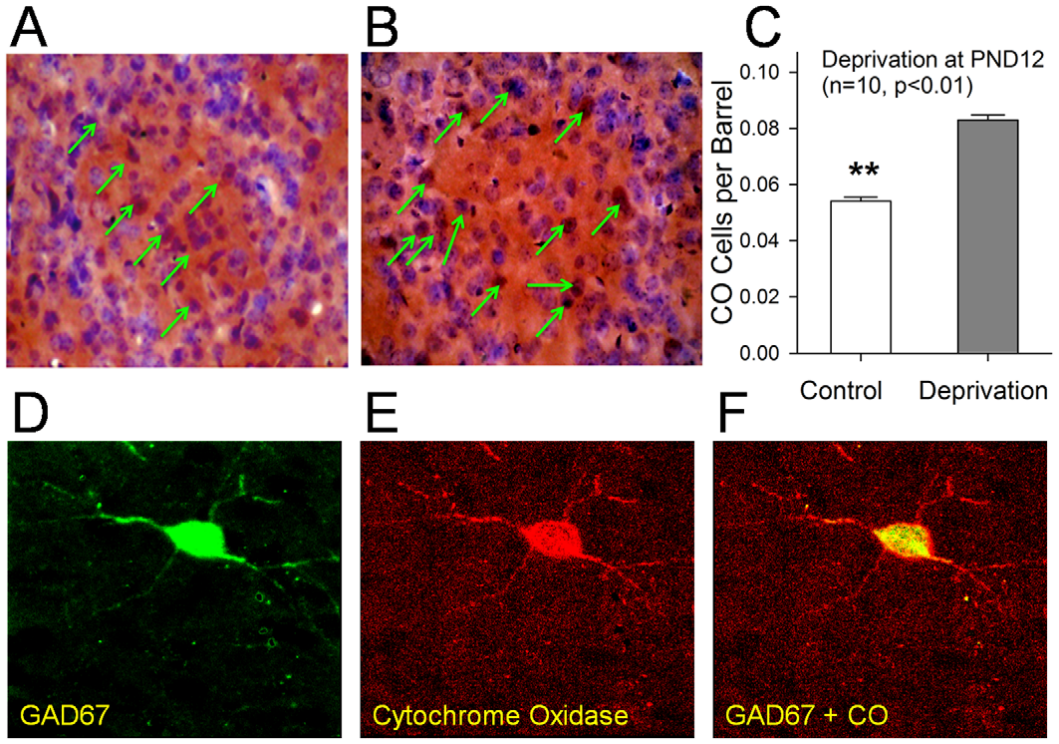
Olfactory deprivation raises the number of Cytochrome Oxidase (CO) positive cells that are GABAergic in mouse barrel cortex. The ratio of CO-positive cells to total cells from each of barrel cross-sections was examined by CO histochemistry and subsequent Nissl's counterstaining, and was estimated based on brown (CO-positive cells) vs. blue (Nissl's) cells. **A**) illustrates a cross-section view of a single barrel from right side (control); and **B**) shows a cross-section view of a single barrel from left side (olfactory deprivation). Green arrows point to CO positive cells. **C**) shows quantitative data in the ratio of CO-positive cells to total per barrel section from right-side (control, white bar) and left-side (olfactory deprivation, gray) in mice (n = 10, p<0.01, **). **D-F**) Barrel CO-positive neurons are GABAergic. Immunocytochemical staining images present glutamate decarboxylase (GAD67) positive neurons (green in **D**), CO-positive cells (red in **E**) and the merged imaging of GAD67-CO cells (**F**) under a laser scanning confocal microscopy.

Fig. 3 shows the effects of olfactory deprivation on the number of total GABAergic cells in barrel cortex. In the cross-section of single barrel, the number of CO-positive cells (brown, pointed by green arrows) vs. Nissl's counterstaining cells (blue) appear lower in control (3A) than olfactory deprivation (3B). The ratios of CO-positive cells to total cells per barrel section on the average are 0.054±0.0015 under control (white bar) and 0.083±0.0018 under olfactory deprivation (gray in Fig. 3C; n = 10, p<0.01). In immunocytochemical staining, GAD67 (Fig. 3D) and CO (Fig. 3E) are co-localized in the same neurons (Fig. 3F). Therefore, olfactory deprivation in mice increases the number of GABAergic neurons vs. total cells in barrel cortex.

**Figure 4:**
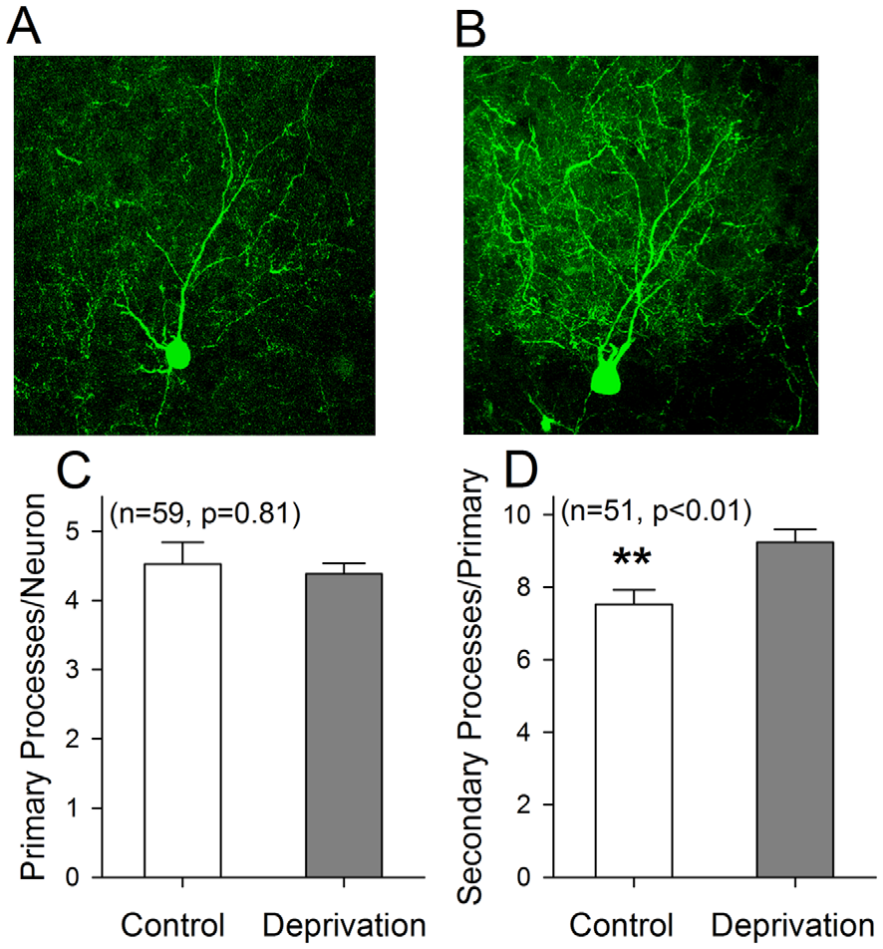
Olfactory deprivation raises process density of barrel GABAergic neurons that are somatostatin-positive and labeled with eGFP. **A**) shows GABAergic neuron and its processes in cross-section of a single barrel from right side (control). **B**) shows GABAergic neuron and its processes in cross-section of single barrel from left side (olfactory deprivation). **C**) shows quantitative data in primary processes from each of GABAergic cells (n = 59) under controls (white bar) and olfactory deprivation (gray, p = 0.81). **D**) shows the secondary processes from each of GABAergic neurons (n = 51) under control (white bar) and olfactory deprivation (gray, p<0.01).

The influences of olfactory deprivation on the process density of GABAergic neurons in barrel cortex were studied in FVB-Tg(GadGFP)4570Swn/J mice (Fig. 4). In the cross-section from single barrels, process density at GABAergic neurons appears lower in control (4A) than olfaction deficit (4B). Fig. 4C illustrates the number of primary processes per GABAergic neuron on the average from each of barrel sections under controls (white bar) vs. olfactory deprivation (gray; n = 59, p = 0.81). Fig. 4D shows that the number of secondary processes per GABAergic neuron is significantly lower under control (white bar) than olfactory deprivation (gray; n = 51, p<0.01). Therefore, olfactory deprivation in mice also increases the fine processes of GABAergic neurons in barrel cortex.

### Olfactory deprivation up-regulates encoding capacity in barrel GABAergic neurons

We investigated the changes in the spike encoding and intrinsic properties of barrel cortical GABAergic neurons in cross-modal plasticity of olfactory deprivation. Their encoding capacity was merited by inter-spike intervals (ISI), and their intrinsic properties include spike threshold potential (Vts) and refractory periods (RP) [29], [30], [31], [32]. In whole-cell recording, ISIs were measured by evoking spikes (depolarization current, 200 ms), and ARPs were done by injecting depolarization pulses (3 ms) into the neurons after each of spikes. Thresholds were a gap between resting membrane potential (Vr) and threshold potential (Vts).

**Figure 5:**
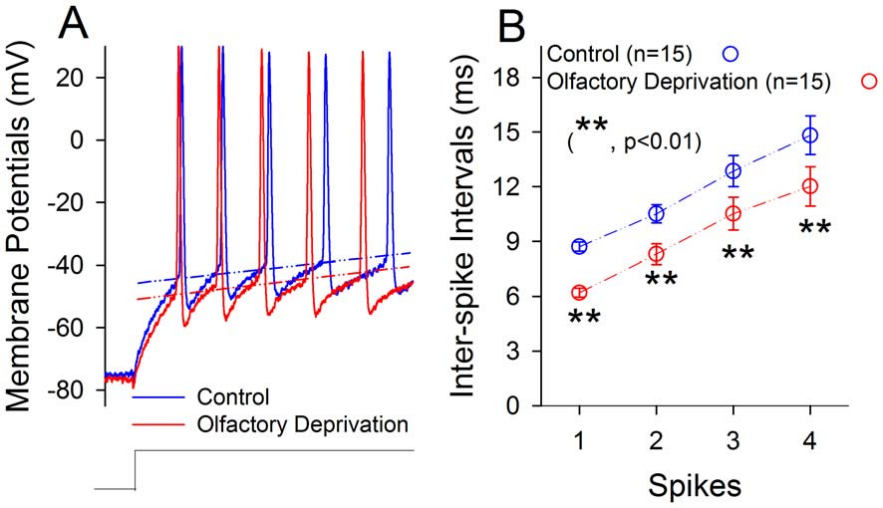
Olfactory deprivation increases the capacity of spike encoding of barrel GABAergic neurons that are somatostatin-positive and labeled with eGFP. Depolarization pulses were injected to evoke sequential spikes. **A**) shows sequential spikes at GABAergic neurons from right-side barrel cortex (control, blue line) and from left-side (olfactory deprivation, red line). Dash lines represent threshold potentials. **B**) shows quantitative data in inter-spike intervals at GABAergic neurons from right-side barrel cortex (control, blue symbols) and from left-side (olfactory deprivation, red symbols; n = 15, p<0.01, **).

Fig. 5 shows the effect of olfactory deprivation on sequential spikes at GABAergic neurons. This manipulation appears to increase the number of spikes in a given time (Fig. 5A). The ISI values of spikes 1∼2 up to 4∼5 are 8.7±0.25, 10.5±0.5, 12.85±0.86 and 14.82±1.06 ms under controls (open symbols in Fig. 5B); and are 6.2±0.25, 8.31±0.56, 10.51±0.9 and 12.0±1.08 ms a week after olfactory deprivation (filled symbols). ISI values for corresponding spikes under two conditions are statistically different (n = 15, p<0.01). Therefore, the capacity of encoding action potentials at barrel GABAergic neurons is enhanced in cross-modal plasticity of olfactory deprivation.

**Figure 6:**
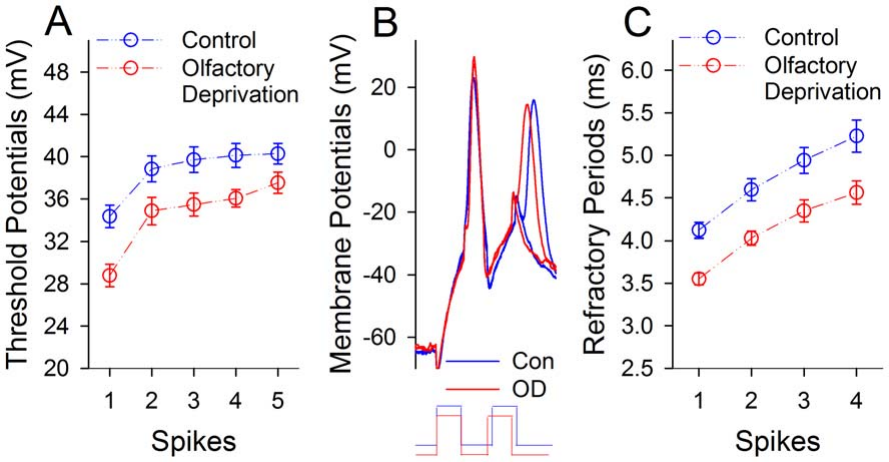
Olfactory deprivation attenuates spike threshold potentials and refractory periods at barrel GFP-GABAergic neurons that are somatostatin-positive. The measurement of spikes' threshold potential was showed in Fig. 5A. **A**) shows the comparison of threshold potentials of sequential spikes at GABAergic neurons from right-side barrel cortex (control, blue symbols) and left-side (olfactory deprivation, red symbols; n = 15, p<0.01). **B**) shows a measurement of refractory periods of spike-1 by two pulses at a GABAergic neuron in right-side barrels (control, blue line) and a cell in left-side (olfactory deprivation, red). **C**) shows spikes' refractory periods at GABAergic cells from right-side barrel cortex (control, blue symbols) and left-side (olfactory deprivation, red symbols; n = 15, p<0.01).

Fig. 6 illustrates the influences of olfactory deficit on Vts and ARPs at barrel cortical GABAergic neurons. The values of Vts-Vr for spikes 1∼5 are 34.34±1.1, 38.83±1.2, 39.7±1.2, 40.11±1.14 and 40.27±1.0 mV under control (open symbols in Fig. 6A); and are 28.8±1.06, 34.85±1.3, 35.5±1.1, 36.1±0.83 and 37.72±1.0 mV under olfactory deficit (filled, n = 15). Vts-Vr values for corresponding spikes under two conditions are statistically different (p<0.01). Furthermore, ARPs at GABAergic neurons appear to be shortened (Fig. 6B). ARP values for spikes 1∼4 are 4.1±0.1, 4.6±0.13, 4.94±0.15 and 5.23±0.2 ms under control (open symbols in Fig. 6C); and are 3.55±0.07, 4.03±0.08, 4.34±0.13 and 4.56±0.14 ms under olfactory deprivation (filled symbols). ARP values for corresponding spikes are statistically different under two conditions (n = 15, p<0.01). Thus, olfactory deprivation reduces threshold potentials and shortens refractory periods to raise the capacity of encoding action potentials at barrel GABAergic neurons.

### Afterhyperpolarization enhances the capacity of encoding sequential spikes

How do we explain the correlation between the hypersensitivity of whisker tactile sensation and upregulation of barrel GABAergic cells after olfactory deprivation? GABAergic neurons may directly take part in encoding tactile sensation with the proportional correlation between tactile sensitivity and their activities. Alternatively, principal neurons encode the sensations; whereas GABAergic neurons increase the sensitivity of principal neurons to input signals. We examined the second hypothesis.

**Figure 7:**
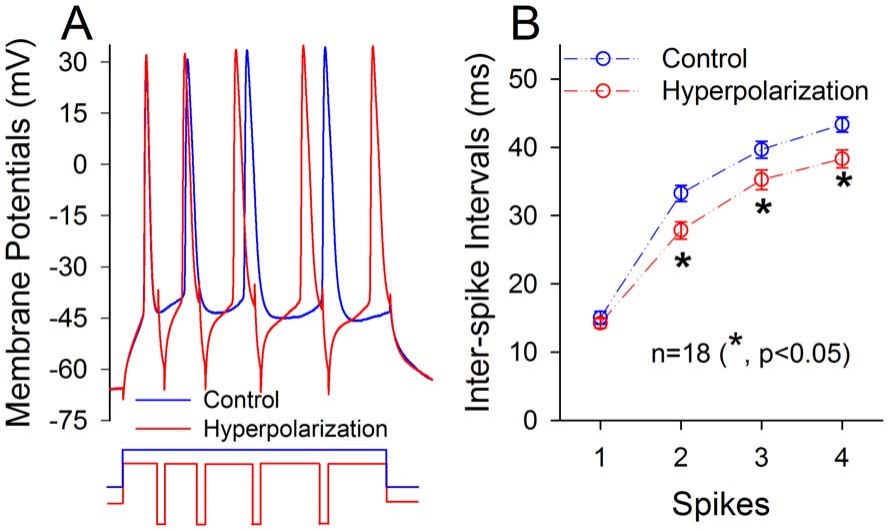
Hyperpolarization enhances the capacity of spike encoding of pyramidal neurons in mouse barrel cortex. Sequential action potentials at barrel cortical pyramidal neurons, which are the target cells of GABAergic neuron in FVB-Tg(GadGFP)4570Swn/J mice, were induced by long-time depolarization pulses or with feedback-inhibition pulses in that hyperpolarization pulses were given after each spike. **A**) shows sequential spikes at pyramidal neurons evoked by long-time depolarization pulse (blue line) and with feedback-inhibition pulses (red line). **B**) illustrates quantitative data in inter-spike intervals of sequential spikes at pyramidal neurons that were evoked by long-time depolarization pulse (blue symbols/line) and inhibitory pulses (red symbols/line; n = 18, *, p<0.05).

GABAergic neurons can be activated after spikes fired at principal neurons via a feedback route [14], [15], [16], [24], which falls into the phase of pyramidal afterhyperpolarization (AHP). To examine the regulation of this inhibitory component in spike encoding, we injected depolarization pulses to evoke spikes and HP pulses (3 ms) immediately after each of spikes to simulate AHP in barrel pyramidal neurons. Fig. 7 shows spike capacity at these neurons with and without giving AHP pulses. Short-term AHP pulses appear to enhance spike encoding (Fig. 7A). Fig. 7B illustrates an analysis of ISI between spikes one and two (ISI_1–2_) up to four and five (ISI_4–5_). Values from ISI_1–2_ to ISI_4–5_ are 15.04±0.95, 33.26±1.17, 39.66±1.23 and 43.34±1.09 ms under control (open symbols); and are 14.23±0.71, 27.83±1.26, 35.24±1.44 and 38.3±1.3 ms under AHP pulses (filled symbols). ISI values for corresponding spikes under the two conditions are statistically different (p<0.01, n = 18), except for ISI_1–2_. Therefore, AHP increases the capacity of encoding sequential spikes, granting our hypothesis that feedback inhibition mediated by GABAergic neurons enhances the function of their target cells (such as principal neurons) in encoding the sensations.

## Discussion

We established a novel model of cross-modal sensory plasticity that olfactory deficit upregulates whisker tactile sensation in mice (Fig. 1), and investigated cell-specific mechanisms in barrel cortex. The hypersensitivity of whisker tactile after the olfactory injury is coming up with the increases in the number of barrel GABAergic neurons (Figs. 2–3), the density of their fine processes (Fig. 4) and the capacity of their encoding digital spikes (Figs. 5–6). AHP mediated by inhibitory inputs onto principal neurons in barrel cortex enhances their capacity of encoding action potentials (Fig. 7). Such changes at the cellular level are embedded into the mechanisms underlying cross-modal sensory plasticity after a loss of olfaction.

It is well known that blindness possesses the enhanced touch and auditory functions for spatial localization, and deaf individuals are alert to visual inputs for their communication [1], [2], [3], [4], [5], [6]. Here, we report a new model of cross-modal plasticity from olfactory loss to whisker tactile sensitization, strengthening a hypothesis that cross-modal sensor plasticity is present in all types of sensations. In cross-modal sensory plasticity, the hypersensitivities in the remained sensory modalities and subsequent sensory substitution in that the information acquired from a sensor modality is used to accomplish the tasks served primarily by another one maintain individuals' awareness to their living environment.

In terms of mechanisms underlying cross-modal sensory plasticity in rodents, the studies show the enlargement of cortical areas for the spared modalities [3], [5], [8], the high expression of certain genes [9], [10] and the rewire/crosswire of neural circuits [7] after the loss of a specific sensation. In studying cell-specific mechanism, we found that the upregulation of GABAergic neurons in barrel cortex is associated with whisker tactile sensitization, and afterhyperpolarization raises encoding capacity at GABA-targeting cells (e.g., principal neurons) in barrel cortex. The viable types of rhythmic activities in central GABAergic cells coordinate the behaviors of principal neurons [15], [16], [17], [24], [25]. These natures in barrel GABAergic neurons and their upregulation may play important role in the sensitization of spared sensory modalities in the cross-modal plasticity.

**Figure 8:**
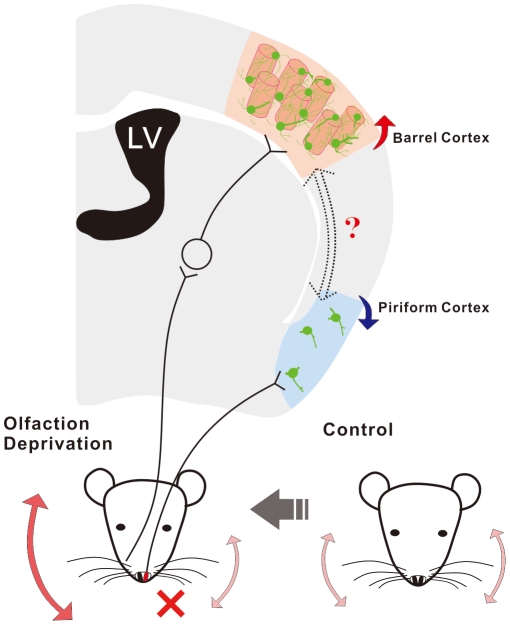
A schematic diagram illustrates cross-modal sensory plasticity from olfaction deprivation to whisker tactile upregulation and the involvement of GABAergic neurons. The left side of olfaction was deficit (red-cross marker), which leads to the down-regulation of piriform cortex (blue arrow). In meantime, whisker tactile sensation in the right side is up-regulated (expanded arrow), compared with the control. This cross-modal plasticity from olfaction to whisker tactile sensation is accompanied by the increases in the number of GABAergic cells (green), the density of their fine processes as well as the functions of encoding digital spikes (red arrow) in the barrels of sensory cortex for whisker tactile (orange columns). How the information about a loss of olfaction from piriform cortex is transmitted to barrel cortex remains to be investigated, as indicated by a question marker.

Regarding the initiation of cross-modal plasticity, how does a cortical area send out the signals about losing a sensory input to other areas for the remained sensory modalities, such as the signals of olfactory deprivation from piriform cortex to barrel cortex in our model? It has been proposed that neural circuits among these areas are rewired or crosswired [7]. In the rewire of neural circuits, there is a formation of new connections between an area of losing sensory input and the areas for other modalities, especially in young age. On the other hand, the crosswire of neural circuits among sensory cortices is physically present and functionally silent, which can be activated by the signals about losing a specific sensation. Either of possibilities remains to be examined (Fig. 8).

Cross-modal sensory plasticity may be initiated by the process that sensory afferents are rewired in cross-modal manner after a loss of sensation. It was observed that the afferents from the remained sensory organs project to a cortical region of losing functional input [3], [5], [7], [8]. Visual stimuli evoke the potentials in the auditory cortex of deaf individuals, and ring stimuli evoke the potentials in blindness's visual cortex. When the afferents from the remained sensory organs project to the cortical area that loses sensory inputs, sensory substitution may form. In the meantime, the signals to the cortices of the remained modalities are weak, which leads to the expansion of these cortices to maintain homeostasis in their sensations and even sensory hypersensitivity. This cross-modal projection of afferents can be explained by the repealing among sensory afferents and the attraction from each of sensory cortices to specific afferents. Signals in Wnts family function to both repeal and attract the extension of axons [33], [34], [35].

It remains to be addressed about molecular mechanisms for the neural circuits to be rewired. This rewire includes the crosswire circuits to be reactivated between sensory cortices and the afferents of the remained modalities to reach a sensory cortex that loses input signals. In our study, it is also to be addressed how GABAergic neurons are up-regulated, whether the morphology and functions of other neurons in barrel cortex are changed after a loss of olfaction, how these never cells coordinately work together to up-regulate whisker tactile sensation. Our finding, the regulation of GABAergic neurons mechanistically for cross-modal sensory plasticity, is an initiative for cross-modal sensory plasticity, a less studied field in neuroscience. A revelation of their mechanisms provides the clues for developing therapeutic approaches to help sensory recovery and substitution.

It may be questioned whether olfactory deprivation on left side affects olfactory pathway on right side, and whether the left side of piriform cortex after olfactory deprivation receives the signals from right side of olfactory pathway, since there is a connection between two sides of olfactory bulbs [36]. The following points do not favor these arguments. The cross-links between olfactory bulbs are not matured until postnatal week two [36], whereas olfaction in our study is deprived at PND 12 (Methods). The deprivation-induced structural defects in olfactory bulbs, such as the loss of glomeruli and olfactory tracts (Figure S2), do not support these cross-links to be developed well. Moreover, when examining piriform cortex on both sides, we observed that the dendrites of GABAergic neurons in the deprived side are less than those in control side (Figure S3), indicating less effect of these cross-links on piriform cortex.

We used the immunocytochemical staining of GAD67 and CO to examine whether all subtypes of GABAergic neurons are involved in cross-modal sensory plasticity (Fig. 3). This datum could be argued by a possibility that CO-positive cells are GABAergic. CO in mitochondria is endogenous metabolic maker for neuronal activity [26], [27]. Highly active GABAergic neurons consume much energy produced from CO-mediated reactions [28]. The neurons with a high CO level are likely GABAergic cells, including somatostain (SOM)- and parvalbumin (PV)-positive neurons [37]. On the other hand, our data at least indicate that the plasticity of SOM-positive GABAergic cells is associated with cross-modal sensory plasticity. As SOM-GABAergic neurons coordinate the activities of large populations of excitatory neurons [11], [12], [13], these coordinated excitatory neurons may upregulate barrel cortex and whisker tactile sensation in mice. We introduce a novel model of cross-modal sensory plasticity from olfactory deficit to whisker tactile hypersensitivity, and present its cell-specific mechanism, i.e., the upregulation of GABAergic neurons that enhance their target-cell's encoding (Fig. 8). Our data provide avenues for therapeutics to benefit sensory recovery and substitution.

## Materials and Methods

### The establishment of cross-modal plasticity relevant to olfactory system

The study and all experiments conducted were fully approved by the Institutional Animal Care Unit Committee in Administration Office of Laboratory Animals Beijing China (B10831). In the model of a loss of olfaction, we have deprived the olfaction permanently by dropping chloroform (40 µl) into the top of unilateral nasal cavity (left side) to injure olfactory epithelium cells in FVB-Tg(GADGFP)4570Swn/J mice (Jackson Lab, USA) at postnatal days (PND) 12. Nasal cavities on right side of these mice were opened for control. One week after this manipulation, the successful deprivation was tested based on the morphological changes of olfactory epithelia (supplementary Fig. S1) and bulb (supplementary Fig. S2). A few of points are given below. Epithelia in left nasal cavity are still functional since mice with olfactory deprivation still have sneezing induced by placing hydrochloride in front of nasal, i.e., trigeminal pathway was not injured. Left olfactory deprivation and right side control are based on the fact that both sides of olfactory system, barrel cortex and whisker behavior are inherently symmetrical [18], [23], [36]. We applied this unilateral olfactory deprivation without examining the physiological loss of olfactory function because mice with bilateral deprivation show an extreme malnutrition and high death rate.

A week after olfactory deprivation, we examined the behavior of whiskers under digital video camera, the intrinsic properties of GFP-labeled GABAergic neurons by whole-cell recordings, the number and morphology of these cells by a laser scanning confocal microscope. We used Cytochrome Oxidase histochemistry plus Nissl's counterstaining and immunocytochemistry to examine a change of total GABAergic neurons. These studies were statistically compared on left side (olfactory loss) vs. right side (control) in the same mice.

### Electrophysiological study

Cerebral cortical slices (400 µm) were prepared from FVB-Tg(Gad- GFP)45704Swn/J mice whose GABAergic neurons express green fluorescent protein (GFP). PND 19–22 mice were anesthetized by injecting chloral hydrate (300 mg/kg) and decapitated by a guillotine. The slices were sectioned with a Vibratome in the modified and oxygenized (95% O_2_ and 5% CO_2_) artificial cerebrospinal fluid (mM: 124 NaCl, 3 KCl, 1.2 NaH_2_PO_4_, 26 NaHCO_3_, 0.5 CaCl_2_, 5 MgSO_4_, 10 dextrose and 5 HEPES; pH 7.35) at 4°C, and were held in normal oxygenated ACSF (mM: 124 NaCl, 3 KCl, 1.2 NaH_2_PO_4_, 26 NaHCO_3_, 2.4 CaCl_2_, 1.3 MgSO_4_, 10 dextrose and 5 HEPES; pH 7.35) 25°C for 1–2 hours. A slice was transferred to a submersion chamber (Warner RC-26G) and perfused with normal ACSF at 31°C for electrophysiological experiments [38].

GFP-labeled GABAergic neurons in layer II-IV of barrel cortex were recorded by whole-cell clamp. These neurons were round/ovary-like soma and tree branch-like processes under DIC optics (Nikon FN-E600), and were identified under fluorescent microscopy by excitation wavelength at 488 nm and emission wavelength at 525 nm.

Action potentials were recorded by MultiClamp-700B and inputted into pClamp9 with 100 Hz sampling rate (Axon Instrument Inc., Foster CA, USA). Transient capacitance was compensated, and output bandwidth filter was 3 kHz. The standard pipette solution contained (mM) 150 K-gluconate, 5 NaCl, 0.4 EGTA, 4 Mg-ATP, 4 Na-phosphocreatine, 0.5 Tris-GTP and 10 HEPES (pH 7.4 adjusted by 2M KOH). Fresh pipette solution was filtered by 0.1 µm centrifuge filter before the use. Pipette solution osmolarity was 295–305 mOsmol, and pipette resistance was 6–8 MΩ.

Neuronal intrinsic properties include refractory periods after each spike, threshold potentials and spike capacity that is measured by inter-spike interval. Absolute refractory period of spike one (ARP1) was measured by injecting two depolarization pulses (3 ms and 5% above threshold) into the neurons, in which pulse one induced spike one at 100% firing probability and inter-pulse intervals were adjusted to have pulse two inducing spike two at 50% firing probability. The duration between spikes 1 and 2 was defined as ARP1 [29]. ARPs of sequential spikes were measured by multiple depolarization pulses (same as above) into the cells. Everyone of action potentials, whose ARPs were measured, was complete in amplitude and just out of relative refractory period of its preceding spikes. By adjusting inter-pulse intervals similar to ARP1 measurement, we read out the durations from the complete spikes to their subsequent spikes of 50% firing probability, i.e., the ARP of sequential spikes (Figure 6B and [29], [30]. In order to measure the properties of sequential spikes, a depolarization pulse (longer than 100 ms) was injected into the GABAergic neurons to induce action potentials (Fig. 5A). Inter-spike intervals (ISI) were the duration between the peaks of the neighboring spikes, and threshold potentials (Vts) are the voltages of firing sequential spikes [29], [30], [39]. The correlation of these parameters is presented in our previous studies [40].

Data were analyzed if the recorded neurons had resting membrane potentials negatively more than −60 mV. The criteria for the acceptation of each experiment also included less than 5% changes in resting membrane potential, spike magnitude, and input/seal resistance. The values of inter-spike intervals (ISI, the index of spike capacity), Vts and ARPs are presented as mean±SE. The comparisons for the data of behaviors, electrophysiology and morphology between groups are done by t-test.

### Morphological study

FVB-Tg(GadGFP)4570Swn/J mice in one week after olfactory deprivation were anesthetized by the intraperitoneal injection of sodium pentobarbital, and were perfused with 4% paraformaldehyde in 0.1 M phosphate buffer solution (PBS) from left ventricle/aorta until the body was rigid. The brains were quickly isolated and fixed in 4% paraformaldehyde PBS for additional 24 hours. In the study of CO histochemistry plus Nissl's counterstaining, the brain tissues were placed in sucrose/PBS with concentrations of 10%, 20% and 30% sequentially until they sank. Cortical tissue was sliced in the cross section of barrel cortex at 20 µm by a freezing microtome. The sections were washed by PBS for 3 times, and stained by CO histochemistry [26]. Subsequent Nissl's counterstaining included that the sections were defatted in xylene, passed in alcohol with gradient concentrations and placed in hematoxylin. Sections were washed, decolorized, dehydrated, air-dried and cover-slipped. The distribution and number of CO-positive neurons (CO higher activity) were observed under conventional optical microscope. In the study of the number of GFP-GABAergic neurons, cortical tissue was sliced in the cross section of barrel cortex at 40 µm by a Vibratome. In the study of the fine structure of GFP-GABAergic neurons, cortical tissue was sliced in the cross section of barrel cortex at 60 µm. The sections were washed in PBS, air-dried and cover-slipped. The images of GFP-GABAergic neurons' structure and number in the cross-sections of barrel cortex were taken under a laser scanning confocal microscopy (Olympus FV-1000, Japan).

In immunocytochemical study of co-localization of CO and glutamate decarboxylase (GAD67), cortical tissue was sliced by a Vibratome for 40 µm. The sections were incubated in monoclonal anti-GAD67 (1∶200) and polyclonal anti-CO (1∶100) antibodies (Sigma, USA) at 4°C with shaking for 24 hours, and then were incubated in FTIC-jointed anti-mouse and red-fluorescent-jointed anti-rabbit (1∶200) antibodies [26], [41]. Images of GAD67 (green) and CO (red) stained neurons in barrel cortex were taken by a laser scanning confocal microscope, in which the parameters of laser beam and PMT were fixed for all experiments.

The number and structures of GABAergic neurons were analyzed by a commercialized software MetaMorph in Meta Imaging Series (ver. 6.1, Universal Imaging Cooperation in Molecular Device). As brain tissues were sliced in series sections, the counting and analysis in cell number and structures were able to be done at least from two sections for each of barrels. The analyzed sections were chosen in a manner of one section from every two in order to prevent the influence of cells that crossed the neighboring sections on the analysis.

### Behavioral studies

Mice sweep whiskers actively to explore their environments and maintain their perception to the world, in which whisking behavior is proportionally associated with whisker tactile sensation [18], [20], [21], [22]. Whisker's tactile sensitivity was tested by free-air whisking and air-puffing induced whisker retraction [23]. Based on data recorded in awaking and restrained mice by digital video camera in high speed, we analyzed the time of whisker sweeping (whisking frequency) and the duration of backward sweeping (retraction duration) induced by puffing air toward whiskers on “deprivation” side versus “control” side. The correlation of whisking strength to tactile sensitivity is based on a rule in physiological reflex, i.e., reaction magnitude is proportional to the sensitivity of sensory system under the condition of constant stimuli.

## Supporting Information

Figure S1 Chloroform (40 µl) dropped into the top of nasal cavity injures olfactory epithelium cells showed by Nissl's staining. Left column presents the images of olfactory epithelia from control (right side of nasal cavity), and right column shows the images of olfactory epithelia from chloroform application to the left side of nasal cavity. Chloroform destroys epithelium layers (40X) and individual cells (100X), include loss of cilia, bulb in cytoplasm and shrink of nuclei.
(3.16 MB TIF)

Figure S2 Chloroform (40 µl) dropped into the top of nasal cavity results in the reduction of olfactory bulb as well as injures the structure of olfactory bulb. Left column presents top and bottom views of mouse brain, in which the left side of olfactory bulb is smaller than the right side when chloroform is dropped into left nasal cavity. Right column shows the cross-sections of olfactory bulbs from the left side (chloroform addition, top panel) and right side (control, bottom panel) stained by Nissl's way. The injury of olfactory epithelia (Fig. 1) further leads to the structural damage of olfactory bulb, especially the loss of glomeruli (peripheral area) and olfactory tracts (central area).
(2.91 MB TIF)

Figure S3 Olfactory deprivation reduces the number of GABAergic cells and their processes in piriform cortex from FVB-Tg(GADGFP)4570Swn/J mice (Jackson Lab, USA) that GABAergic neurons are genetically labeled with eGFP. Left column shows the images of piriform cortex from control (right side), and right column shows the images of priform cortex from chloroform application to left side of nasal cavity. Compared to control (left column), olfactory deprivation reduces the number of GABAergic neurons (top panels, 10X) and their processes (bottom panels, 40X).
(1.42 MB TIF)
