# Supplementary figures and images for: Upregulation of Barrel GABAergic Neurons Is Associated with Cross-Modal Plasticity in Olfactory Deficit
Source: PLoS One. 2010 Oct 29;5(10):e13736. doi: 10.1371/journal.pone.0013736 (PMC2966404; doi:10.1371/journal.pone.0013736)

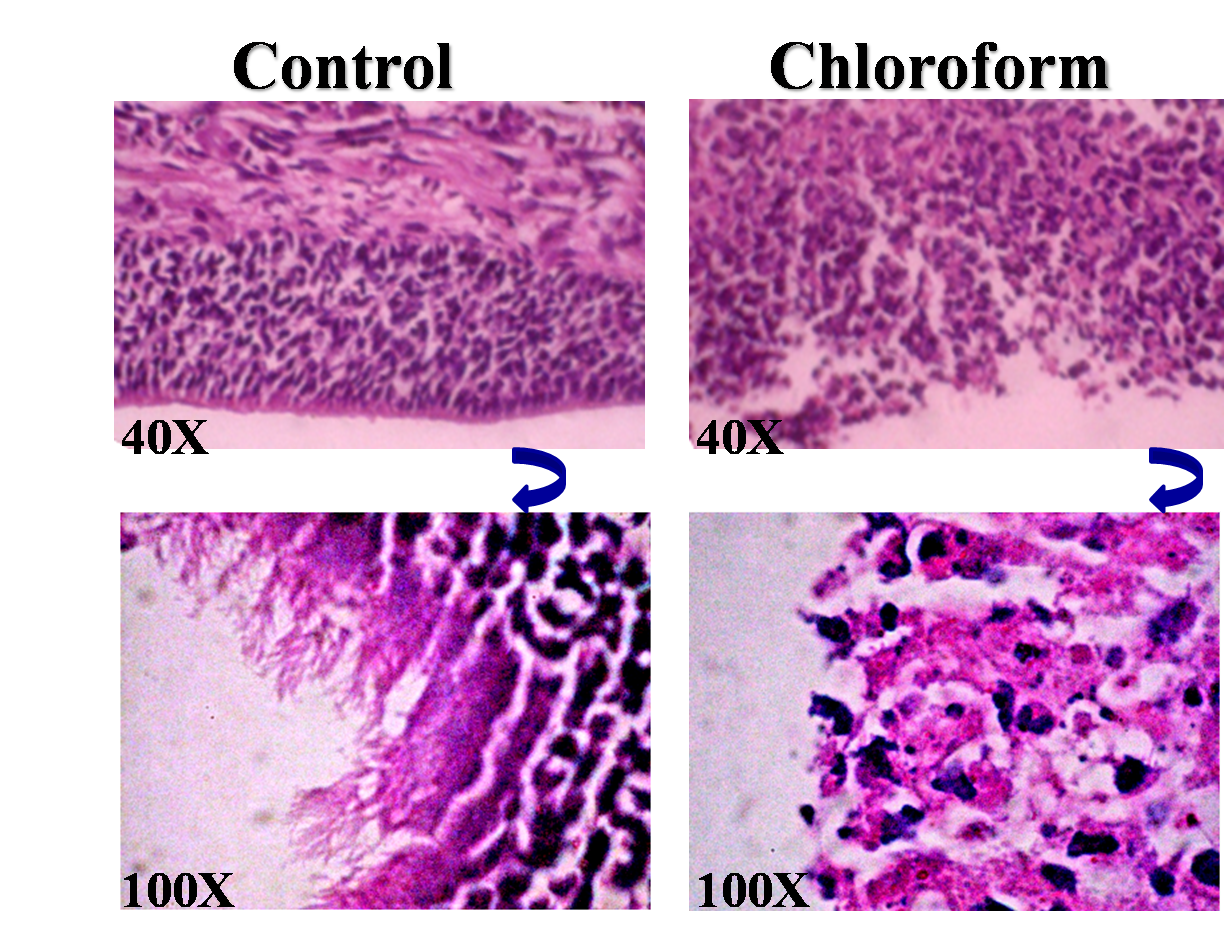

Supplement: Figure S1 — Chloroform (40 µl) dropped into the top of nasal cavity injures olfactory epithelium cells showed by Nissl's staining. Left column presents the images of olfactory epithelia from control (right side of nasal cavity), and right column shows the images of olfactory epithelia from chloroform application to the left side of nasal cavity. Chloroform destroys epithelium layers (40X) and individual cells (100X), include loss of cilia, bulb in cytoplasm and shrink of nuclei. (3.16 MB TIF) [file pone.0013736.s001.tif]

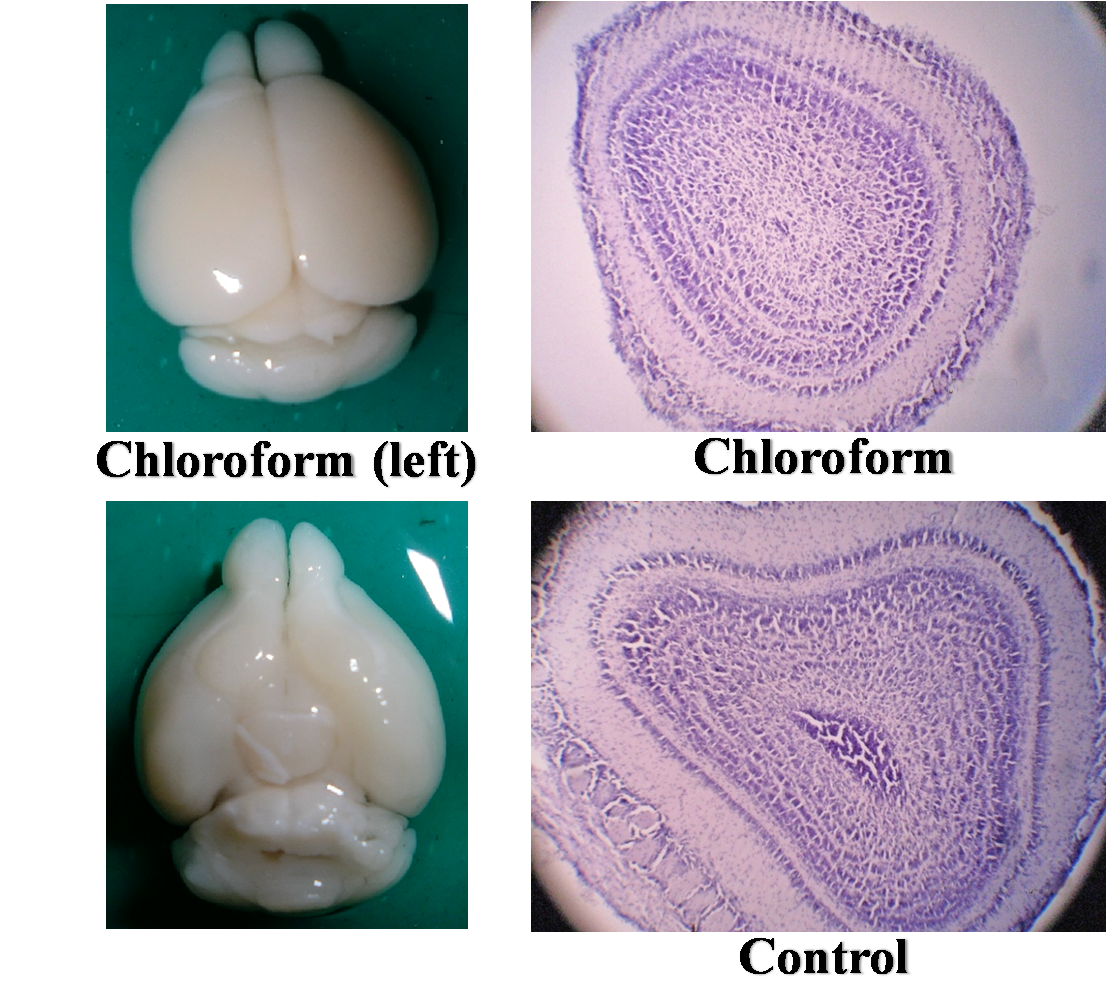

Supplement: Figure S2 — Chloroform (40 µl) dropped into the top of nasal cavity results in the reduction of olfactory bulb as well as injures the structure of olfactory bulb. Left column presents top and bottom views of mouse brain, in which the left side of olfactory bulb is smaller than the right side when chloroform is dropped into left nasal cavity. Right column shows the cross-sections of olfactory bulbs from the left side (chloroform addition, top panel) and right side (control, bottom panel) stained by Nissl's way. The injury of olfactory epithelia (Fig. 1) further leads to the structural damage of olfactory bulb, especially the loss of glomeruli (peripheral area) and olfactory tracts (central area). (2.91 MB TIF) [file pone.0013736.s002.tif]

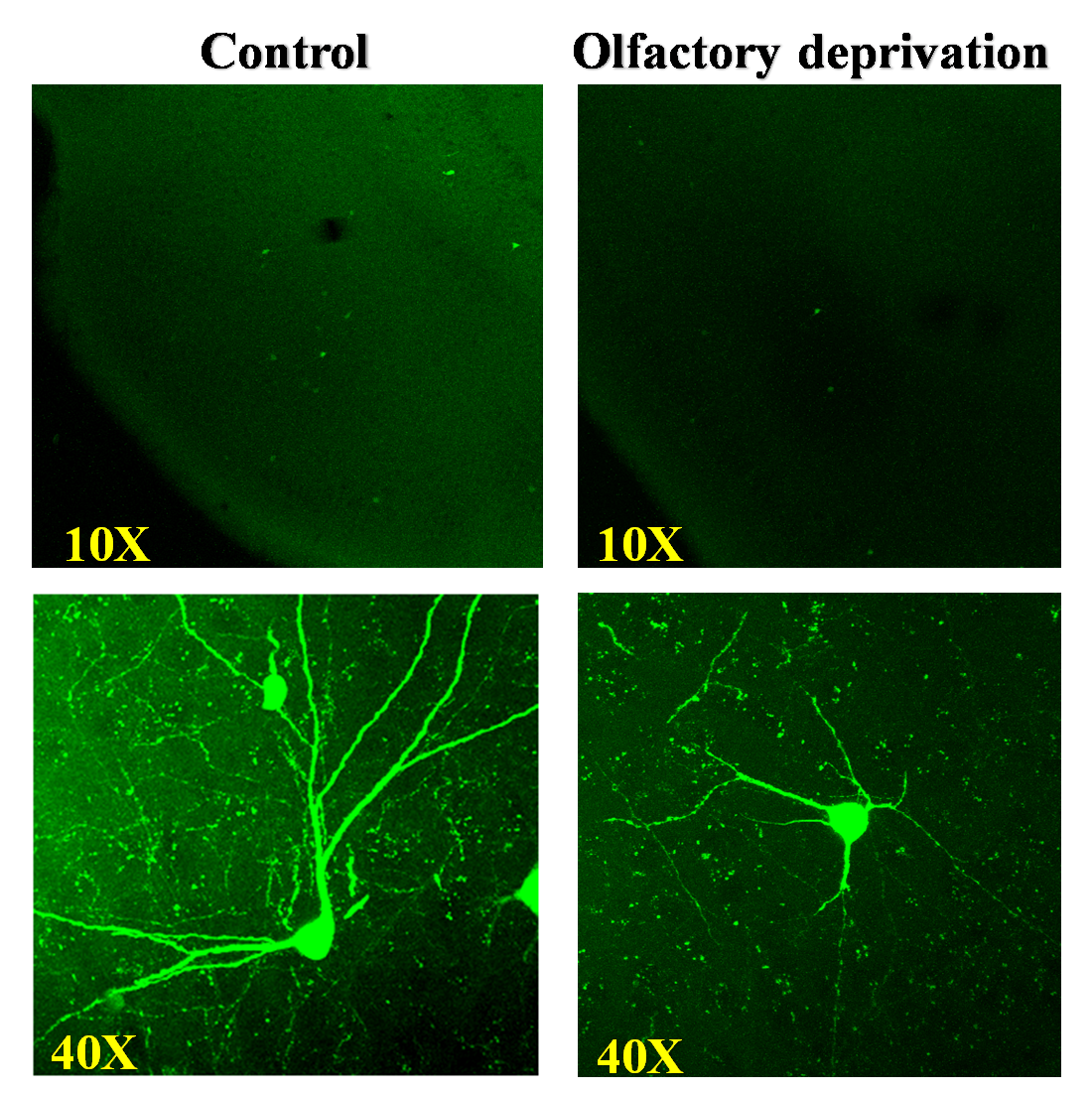

Supplement: Figure S3 — Olfactory deprivation reduces the number of GABAergic cells and their processes in piriform cortex from FVB-Tg(GADGFP)4570Swn/J mice (Jackson Lab, USA) that GABAergic neurons are genetically labeled with eGFP. Left column shows the images of piriform cortex from control (right side), and right column shows the images of priform cortex from chloroform application to left side of nasal cavity. Compared to control (left column), olfactory deprivation reduces the number of GABAergic neurons (top panels, 10X) and their processes (bottom panels, 40X). (1.42 MB TIF) [file pone.0013736.s003.tif]
